# Dectin-1/TLR2 and NOD2 Agonists Render Dendritic Cells Susceptible to Infection by X4-Using HIV-1 and Promote *cis*-Infection of CD4^+^ T Cells

**DOI:** 10.1371/journal.pone.0067735

**Published:** 2013-07-02

**Authors:** Sandra C. Côté, Audrey Plante, Mélanie R. Tardif, Michel J. Tremblay

**Affiliations:** 1 Axe des Maladies Infectieuses et Immunitaires, Centre Hospitalier Universitaire de Québec-Pavillon CHUL, Québec, Canada; 2 Département de Microbiologie-Infectiologie et Immunologie, Faculté de médecine, Université Laval, Québec, Canada; New York University, United States of America

## Abstract

HIV-1 pathogenesis is intimately linked with microbial infections and innate immunity during all stages of the disease. While the impact of microbial-derived products in transmission of R5-using virus to CD4^+^ T cells by dendritic cells (DCs) has been addressed before, very limited data are available on the effect of such compounds on DC-mediated dissemination of X4-tropic variant. Here, we provide evidence that treatment of DCs with dectin-1/TLR2 and NOD2 ligands increases *cis*-infection of autologous CD4^+^ T cells by X4-using virus. This phenomenon is most likely associated with an enhanced permissiveness of DCs to productive infection with X4 virus, which is linked to increased surface expression of CXCR4 and the acquisition of a maturation profile by DCs. The ensuing DC maturation enhances susceptibility of CD4^+^ T cells to productive infection with HIV-1. This study highlights the crucial role of DCs at different stages of HIV-1 infection and particularly in spreading of viral strains displaying a X4 phenotype.

## Introduction

Early after mucosal exposure, human immunodeficiency virus type-1 (HIV-1) crosses the epithelial cell barrier and reaches the lamina propria where it is eventually captured by resident sentinel immune cells. The precise mechanism(s) by which the virus activates innate immunity yet remains unclear, but perturbation of the mucosal barrier by inflammation and/or the presence of a pre-existing infection enhance the HIV-1 transmission process. For instance, epidemiologic studies demonstrated a strong correlation between HIV-1 acquisition and the presence of sexually transmitted diseases causing mucosal ulcers, such as herpes simplex and syphilis [1], [2], and of non-ulcerative infections, such as gonorrhoea, chlamydia or vaginosis [3], [4]. It has been proposed that the disruption of epithelial integrity facilitates translocation of the residing microbiota and HIV-1 to the lamina propria and promotes inflammation.

Strategically located under the mucosal barrier, immature dendritic cells (iDCs) sense environmental antigens, pathogens and microbial-derived products upon their translocation across the epithelial cell layer. Exposure to microbial-derived products promotes maturation of iDCs and their subsequent migration to lymph nodes where they can present antigen to resting CD4^+^ T cells and activate adaptive immunity. It is known that iDCs can detect microbial-derived products better known as pathogen-associated molecular patterns (PAMPs) via diverse pattern-recognition receptors (PRRs), such as Toll-like receptors (TLRs), NOD-like receptors and C-type lectin-like receptors (CLRs). It has been shown that iDCs express high surface levels of TLR1/2, TLR2/6, TLR5 and the CLR dectin-1 and high amounts of intracellular receptors such as TLR3, TLR8, TLR9, NOD1 and NOD2 (reviewed in [5], [6]). The nature of the stimuli, the type of innate receptor engaged and the cytokinic environment modulate the DC maturation program, which in turn drives polarization of naïve CD4^+^ T cells toward a Th1, Th2 or Th17 pattern [7], [8].

Previous observations indicate that CCR5-tropic strains of HIV-1 (i.e. also called R5) can productively infect iDCs, but viral replication is much less effective than in effector CCR5^+^ CD4^+^ T cells. Nevertheless, such virus-carrying iDCs can facilitate HIV-1 spreading through *cis*-infection (i.e. direct infection) of susceptible effector CCR5^+^ CD4^+^ T cells located deeply within the mucosa. It has been also shown that both R5- and CXCR4 (X4)-using HIV-1 variants can also exploit DCs by using them as a Trojan horse to facilitate viral propagation and evade antiviral immunity [9], [10], [11]. This phenomenon is termed *trans*-infection and is mostly orchestrated once DCs are activated and migrate to lymphoid tissues. Virus transfer takes place after synapse formation with surrounding effector CD4^+^ T cells. The magnitude of viral replication is then closely linked to the T-cell activation state [12]. In naïve CD4^+^ T cells, which account for approximately 65 to 85% of all CD4^+^ T cells found in peripheral blood and secondary lymphoid organs (SLOs), HIV-1 replication is mostly inefficient, but the virus can remain latent in central memory CD4^+^ T cells and be reactivated depending on the surrounding microenvironment [13], [14], [15]. In effector CD4^+^ T cells, particularly Th1 and Th17 subsets, viral replication is massive and ultimately leads to a progressive T-cell depletion [16], [17].

During the course of infection, about 50% of HIV-1-infected subjects harbor X4-tropic viral strains, in addition to R5-using variants (clade B), and this emergence leads to a rise of viremia and disease progression [18], [19]. Several hypotheses have been proposed to explain the mechanism(s) underlying the coreceptor switch (i.e. R5 to X4), but this phenomenon still remains enigmatic (reviewed in [20], [21]). It has been postulated that the thymus, which is the primary site of T lymphopoiesis during early life, plays a dominant role in the emergence and amplification of X4 virus in pediatric HIV-1 infection [22]. Moreover, it has been suggested that a high CXCR4 density on the surface of CD4^+^ T cells during the course of HIV-1 infection favors the emergence of X4 virus isolates [23], [24]. Such X4 virions evolve and expand in certain tissue compartments, particularly in lymphoid organs where the number of CXCR4^+^/CCR5^-^ CD4^+^ T cells is high. A recent work performed by Wuze Ren and colleagues revealed that, in macaques infected with both R5 and X4 HIV-1, no X4 variants were found in the gut, peripheral tissues, bone marrow or spleen, even at 13 weeks post-infection, but they were detected in peripheral lymph nodes [25]. Taken together, these data suggest that lymphoid tissues enriched with CXCR4^+^/CCR5^–^ CD4^+^ T cells can be considered as privileged sites supporting the emergence and the expansion of X4-using viruses. However, CXCR4^+^/CCR5^–^ CD4^+^ T cells belong to the naïve or central memory subset in which HIV-1 cannot efficiently replicate, thus additional events are required to support expansion of X4 virus in such a specialized microenvironment.

Results from previous studies have revealed contradictory effects about involvement of microbial-derived products in transmission of R5-using isolates of HIV-1 to effector CD4^+^ T cells by mature DCs (mDCs) [26], [27]. For example, it was suggested that this process was influenced by the nature of the receptor engaged on the surface of mDCs [26], [27]. However, there is still very limited information about the possible contribution of PAMP-induced DC maturation on transmission of X4-using HIV-1 to CD4^+^ T cells. In this present study, we demonstrate that bacterial- and yeast-derived products render DCs susceptible to infection by X4-using HIV-1. Moreover, we show that these PAMP-treated DCs promote *cis*-infection of autologus CD4^+^ T cells by establishing an intracellular milieu more permissive to productive HIV-1 infection.

## Results

All the experiments described hereinafter made use of a variety of highly purified PAMPs rigorously chosen based on their ability to engage either a single PRR or a very restricted number of them. In order to reflect microbial products likely to be encountered by DCs in the context of of HIV-1 infection, we selected the following bacterial-derived molecules: *Escherichia coli* 0111:B4-derived ultrapure lipopolysaccharide (LPS) (TLR4 agonist) in which the molecule was treated with successive enzymatic hydrolysis steps to remove bacterial lipoproteins activating TLR2 that are largely present in standard LPS; *Salmonella typhimurium*-derived ultrapure flagellin (TLR5 ligand); PGN-SAndi (NOD2 agonist), a peptidoglycan from *Saphylococcus aureus* treated to remove lipoproteins activating TLR2; and Pam3Csk4, a synthetic lipopeptide mimicking bacterial lipoproteins (TLR2 ligand). In addition to these, two yeast-derived products were tested: namely zymosan, which is a *Saccharomyces cerevisiae* cell wall preparation (dectin-1 and TLR2 agonist), and a depleted form of zymosan (D-zymosan), which has been treated with hot alkali to remove all of its TLR2-stimulating properties, and thus only activates dectin-1. Finally, polyinosinic-polycytidylic acid (polyI:C), a synthetic analog of double-stranded RNA, was chosen to mimic viral infection (TLR3 agonist, although it can also stimulate RIG-I and MDA5).

### Different PAMPs Elicit Distinct Modulatory Effects on HIV-1 Replication in DC-T Cell Co-cultures

**Figure 1:**
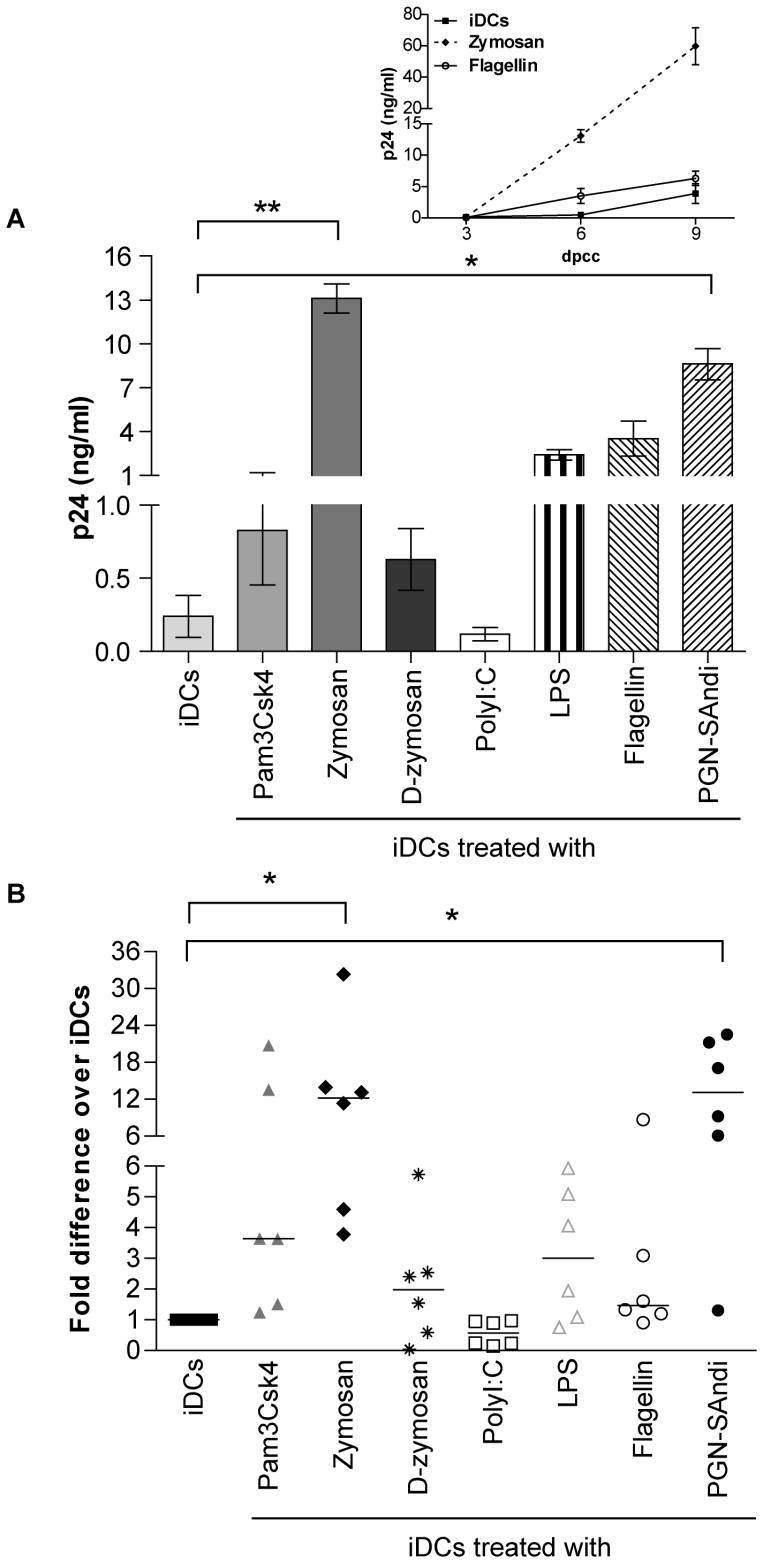
Replication of X4 virus in DC-T cell co-cultures is influenced by the nature of PAMPs. iDCs were first either left untreated or treated for 24 hours with the listed PAMPs. Next, cells were exposed to X4-using NL4-3 for 1 hour at 37°C before initiation of a co-culture with autologous resting CD4^+^ T cells. Cell-free supernatants were harvested at 3, 6 and 9 days after initiation of the co-culture and the viral content was assessed by performing a p24 ELISA test. (A) Data shown represent the means ± SEM of quadruplicate samples from one representative donor out of six at day 6 after initiation of the co-culture (days post-coculture/dpcc). The small insert shows kinetics of virus production for this representative donor (only iDCs either left untreated or treated with flagellin or zymosan are illustrated). (B) This panel illustrates data for all six independent donors. Each point represents the mean of quadruplicate samples for each donor. The horizontal line represents median results of all donors. Asterisks denote statistically significant data (*: p<0.05; **: p<0.01).

We first investigated the ability of DCs, which were induced to mature with the above-listed PAMPs, to transmit X4-using virus to autologous CD4^+^ T cells that are in a resting state at the start of the co-culture experiment. It should be noted that we intentionally used the latter cell population because the majority of circulating CD4^+^ T cells are in a resting state. We did perform some preliminary studies where replication of X4 virus in DC-T cell co-cultures was monitored using four different doses of each PAMP (data not shown). Data from this series of investigations has allowed us to choose a single effective concentration of each PAMP that was used throughout our work (i.e. 1 µg/ml for Pam3Csk4, polyI:C, flagellin and PGN-SAndi; 5 µg/ml for zymosan and D-zymosan; and 10 ng/ml for LPS). As depicted in Figure 1, virus propagation in co-cultures made of PAMP-treated DCs and autologous CD4^+^ T cells is differently affected by the divergent microbial-derived products. For example, while engagement of TLR3 by polyI:C reduces virus production, as compared to untreated iDCs, the majority of the other PAMPs tested enhance replication of X4 virus in such co-cultured cells. However, a statistically significant augmentation in virus production was seen only with zymosan and PGN-Sandi. Similar studies were carried out with a R5-tropic variant and a less pronounced increase in virus production was seen upon treatment with only two of the tested agonists (i.e. 2- and 3-fold increase with Pam3Csk4 and zymosan, respectively) (Figure S1). Treatment with D-zymosan and PGN-SAndi had minimal effect, whereas replication of R5 virus in DC-T cell co-cultures was slightly decreased by LPS and almost completely abrogated by polyI:C.

The putative mechanisms that can affect propagation of X4 virions in DC-T cell co-cultures include for example the maturation stage of DCs, the activation status of CD4^+^ T cells and the formation of a virological synapse (a phenomenon favouring cell-to-cell transfer of HIV-1). Additional studies were thus carried out to shed light on this issue.

### Different PAMPs Mediate Distinctive DC Maturation Profiles

Considering our previous observations suggesting that the nature of microbial-derived molecules used to drive maturation of DCs differently influences HIV-1 production in DC-T cell co-cultures, we first investigated whether it affects the maturation stage of DCs. To this end, iDCs were exposed to the different PAMPs, and we monitored several key features of mDCs such as morphological aspect, expression of specific surface markers, cytokine expression and release of type-I interferon (IFN).

We found that iDCs treated with the studied PAMPs acquired typical morphological features of mature DCs as determined by phase-contrast microscopy (data not shown). Indeed, all the PAMPs tested enhanced cell adhesion at the bottom of the plate and most of them induced the formation of cytoplasmic projections characterizing DCs with a mature phenotype (data not shown). Moreover, thin and elongated DCs were found in samples treated with PGN-SAndi, zymosan and D-zymosan, while cell clusters were observed in the presence of Pam3Csk4 (data not shown).

**Figure 2:**
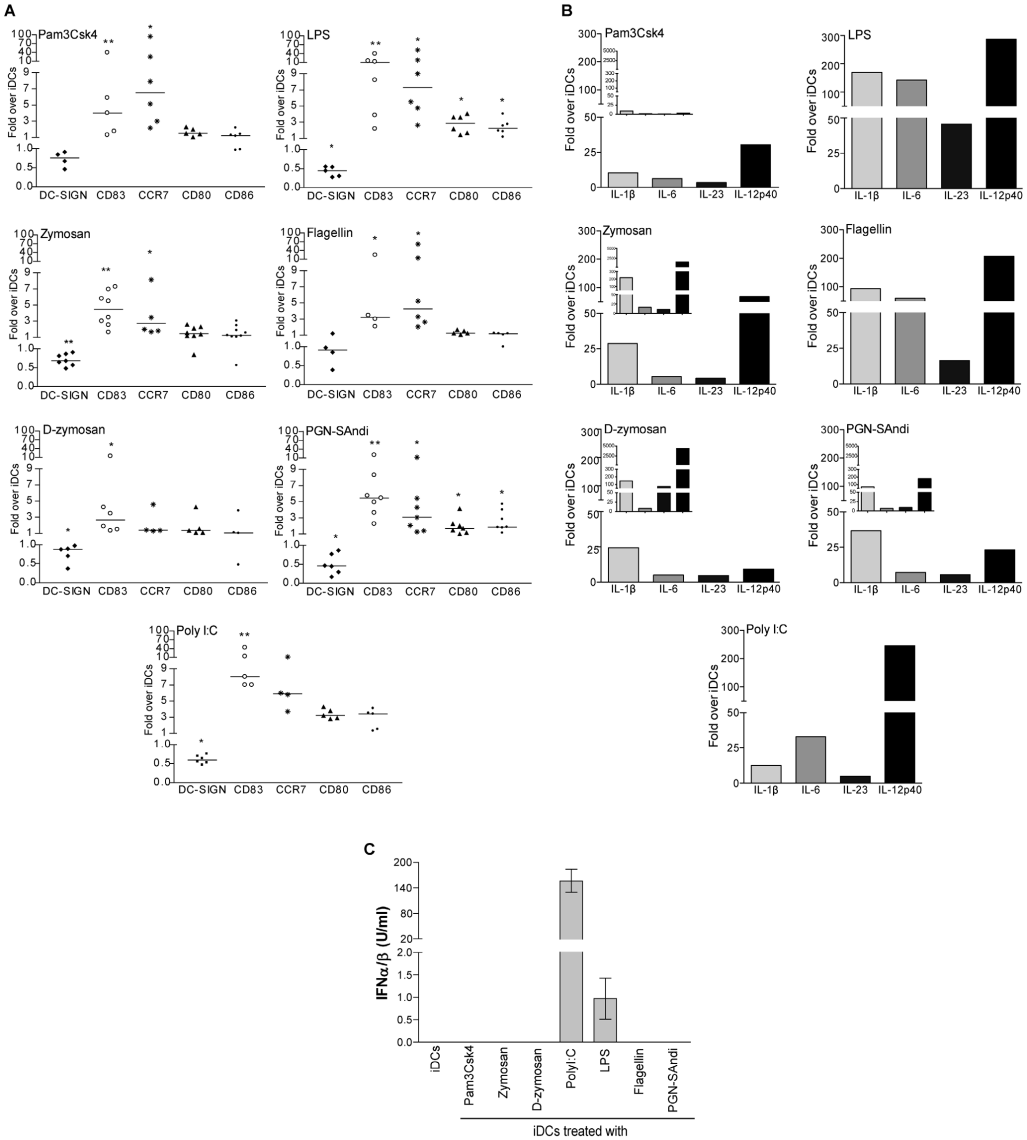
The nature of PAMPs modulates both maturation profile and cytokine expression pattern of DCs. (A) iDCs were either left untreated or treated for 72 hours with the indicated PAMPs. Afterwards, cell surface expression of DC-SIGN, CD83, CCR7, CD80 and CD86 was evaluated by flow cytometry. Data are expressed as the percentage of positive cells multiplied by the mean fluorescence intensity for the PAMP-stimulated DCs compared to untreated DCs (i.e iDCs). Each point represents a different donor. The horizontal line represents the median of the values for all donors. Asterisks denote statistically significant data (*: p<0.05; **: p<0.01). (B) iDCs were treated with the indicated PAMPs for 4 or 24 hours (small inserts) and total RNA was extracted. The variation of IL-1β, IL-6, IL-23 and IL-12p40 mRNA levels was evaluated by real-time PCR. After normalization on 18S ribosomal RNA subunit, the variation of mRNA levels for each cytokine was compared to that of untreated cells. Data depicted represent results obtained from one representative donor out of four. (C) iDCs were either left untreated or stimulated for 6 hours with the listed microbial-derived products. Cell-free supernatants were harvested and the levels of IFNα/β were quantified using HEK-Blue™ IFNα/β cells. Data shown represent the mean ± standard deviations of quadruplicate samples from one donor representative of two.

Such PAMP-treated cells also exhibited differences with respect to expression of some specific surface markers following a treatment with the tested PRR agonists. For example, cells exposed to polyI:C, LPS and PGN-SAndi expressed about 50% less DC-SIGN, an antigen capture receptor characterizing iDCs, as compared to cells left untreated with PAMPs (Figure 2A) (percentage of positive cells as well as mean fluorescence intensity/MFI for the tested surface markers are depicted in Figure S2). The decrease was less pronounced with Pam3Csk4, D-zymosan and zymosan, while flagellin does not alter the level of DC-SIGN expression. The intensity of CD83, which is considered as one of the best marker for DC maturation, also varied depending of PAMPs, but was increased in all instances. The highest increase occurred in cells treated with LPS and polyI:C (at least an 8-fold increase), followed by PGN-SAndi, zymosan and Pam3Csk4 (4- to 8-fold increase), and finally by flagellin and D-zymosan (lower than a 4-fold increase). Knowing the crucial role played by the chemokine receptor CCR7 in the migration of DCs to SLOs, we analyzed also expression of this surface molecule. We found that LPS, polyI:C and Pam3Csk4 trigger the most significant increase in surface expression of CCR7 (greater than a 5-fold increase). All the other microbial stimuli increased CCR7 at least by a factor of 3, with the exception of D-zymosan, where CCR7 remained unchanged compared to untreated iDCs. Therefore, most PAMP-treated DCs acquire characteristics required for their migration to lymph nodes. Once in SLOs, such mDCs may interact with naïve and/or central memory CD4^+^ T cells to initiate antigen recognition. This phenomenon is known to rely on expression of co-stimulatory molecules, such as CD80 and CD86. Hence, we monitored the presence of these molecules and, surprisingly, only LPS-, PGN-SAndi- and polyI:C-treated DCs showed a significantly higher expression of the two molecules.

During the formation of an immunological synapse, DCs secrete cytokines modulating T-cell activation and the subsequent switch to various Th profiles. IL-12p70 is known to drive the switch to the Th1 subset, whereas IL-6, IL-23 and TGF-β direct the cells toward the Th17 phenotype [28]. Both of these Th subpopulations are known to be productively infected with HIV-1 [29]. In order to characterize the cytokine pattern induced by the studied PAMPs, iDCs were treated with microbial stimuli for either 4 or 24 hours and Th1- and Th17-specific cytokine mRNA levels were assessed by real-time PCR. Results illustrated in Figure 2B show high differences between PAMPs. Indeed, treatment of iDCs with LPS and flagellin induced production of higher amounts of IL-1β, IL-6, IL-23p19 and IL-12p40 mRNAs compared to untreated cells. These changes are transient since their mRNA levels decrease after 24 hours of treatment (data not shown). For zymosan-, D-zymosan- and PGN-SAndi-treated DCs, the kinetics was slower. In fact, more variations of cytokine mRNAs were seen after 24 hours of treatment for IL-1β, IL-23p19 and IL-12p40. For these last two PAMPs, IL-6 mRNA levels remained mainly stable over time and only 5-fold more elevated than those seen in untreated iDCs. However, in DCs stimulated with zymosan, the mRNA of IL-6 increased and reached 20 folds compared to iDCs. In Pam3Csk4-treated DCs, we detected low variations of cytokine mRNAs compared to untreated iDCs for the two time points tested (i.e. 4 and 24 hours). TGF-β mRNA was also measured and results showed that untreated iDCs and PAMP-exposed DCs contained an elevated level of this transcript (data not shown). Since the Th1-driving cytokine IL-12p70 is a heterodimer complex formed by IL-12p40 and Il-12p35, we next investigated whether PAMP-treated DCs could express IL-12p70. The IL-12p35 subunit mRNA was however only detectable in polyI:C-treated DCs and more weakly in LPS-stimulated cells (data not shown). To further demonstrate that some PAMP-exposed DCs adopt a Th1-polarizing function, the release of functional type-I IFN was measured at 6 hours post-exposure using HEK-blue^TM^IFNα/β indicator cells. As depicted in Figure 2C, type-I IFN was produced in a significant manner by polyI:C-treated DCs. Given that type-I IFN displays some antiviral properties, we postulate that the reduced virus production seen in polyI:C-treated DCs (Figure 1) is caused by this soluble factor. Hence, this PRR agonist was removed from our subsequent experiments. Overall, our results show that most of the studied PAMPs promote either an intermediate or full maturation process of human DCs.

### PAMP-treated DCs Sensitize CD4^+^ T Cells and Lead to a Productive *cis*-infection by X4 Virus

Previous published observations indicate that resting CD4^+^ T cells are refractory to productive infection with HIV-1 and the mechanism(s) at play and its cellular mediators have still remained elusive. Nevertheless, it has been hypothesized that susceptibility to HIV-1 infection is under the control of the host cell machinery [30]. In an attempt to compare the ability of DCs once treated with PAMPs to sensitize autologous CD4^+^ T cells that are resting before initiation of the co-culture to productive HIV-1 infection, we first measured the presence of activation markers on the surface of CD4^+^ T cells. We did not observe any significant enhancement of expression of several activation markers (i.e. ICAM-1, CD25, CD69 and HLA-DR) (data not shown). We also measured CCR5 expression and no significant differences were observed, even after 7 days of co-culture (data not shown). We next evaluated the ability of PAMP-treated DCs to induce proliferation of CD4^+^ T cells and found no evidence of cell proliferation as monitored by CFSE staining (data not shown), which suggest that the PAMP-mediated maturation of DCs is not sufficient *per se* to fully activate resting CD4^+^ T cells.

**Figure 3:**
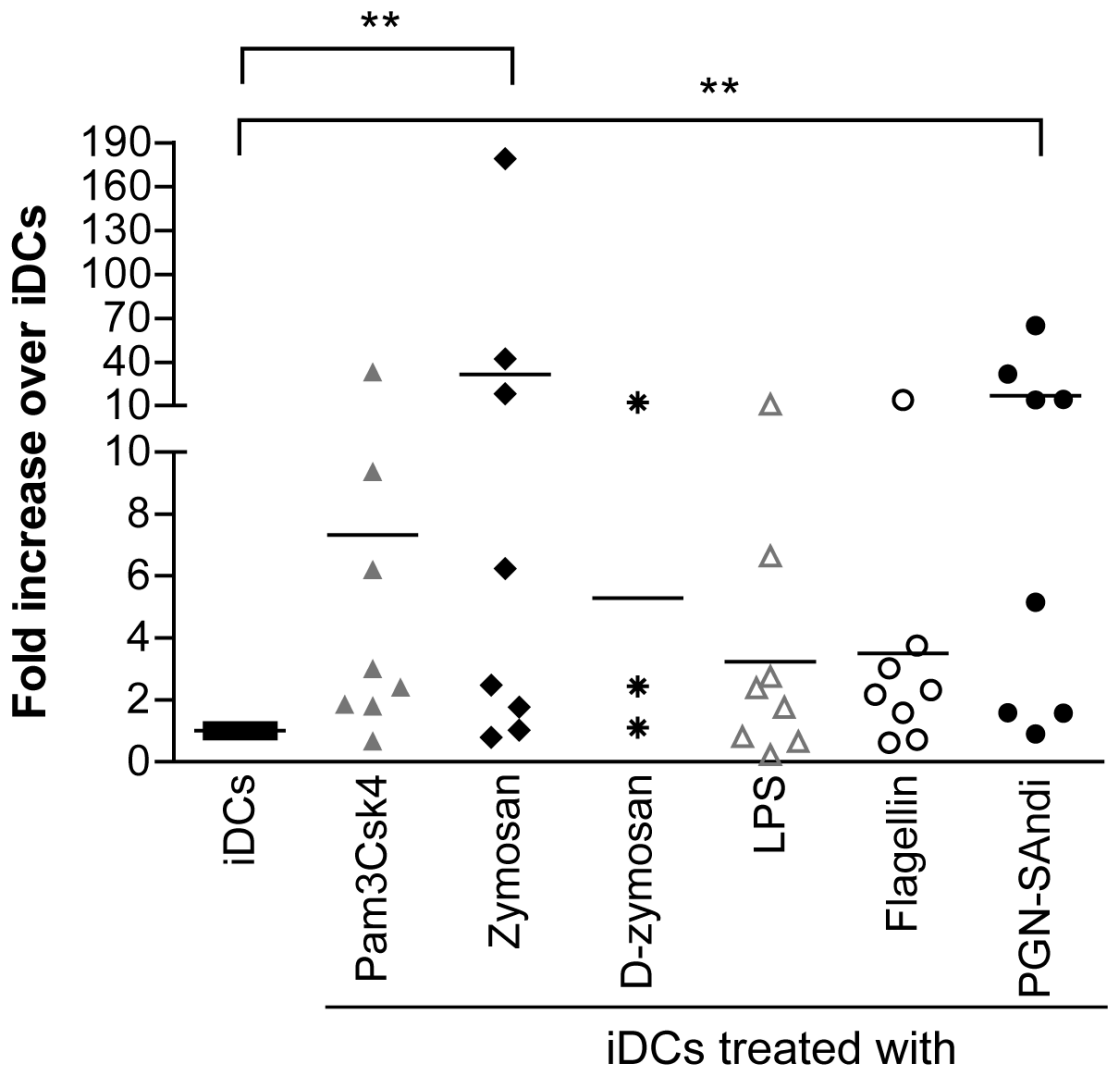
PAMP-treated DCs sensitize resting CD4^+^ T cells to productive HIV-1 infection. iDCs were either left untreated or treated with the indicated PAMPs for 48 hours. Autologous resting CD4^+^ T cells were infected with NL4-3 for 48 hours, extensively washed and co-cultured with iDCs or PAMP-stimulated DCs. Thereafter, cell-free supernatants were collected at day 6 following initiation of the co-culture and the p24 content was assessed by ELISA. Each point represents the mean of quadruplicate samples for one donor. The horizontal line represents mean result of all donors. Asterisks denote statistically significant data (*: p<0.05; **: p<0.01).

We next wanted to determine whether an interaction between PAMP-treated DCs and autologous resting CD4^+^ T cells previously inoculated with X4 virus can promote HIV-1 replication in the latter cell type. Our results showed that a contact between mature DCs and resting CD4^+^ T cells is nonetheless a determining factor to establish a proper microenvironment for productive infection with X4 virions. Indeed, our results indicate that a much more efficient HIV-1 production is seen when PAMP-treated DCs (i.e. exposed to zymosan and PGN-Sandi) are incubated together with autologous resting CD4^+^ T cells that were first exposed to X4 virus prior to initiation of the co-culture (Figure 3).

**Figure 4:**
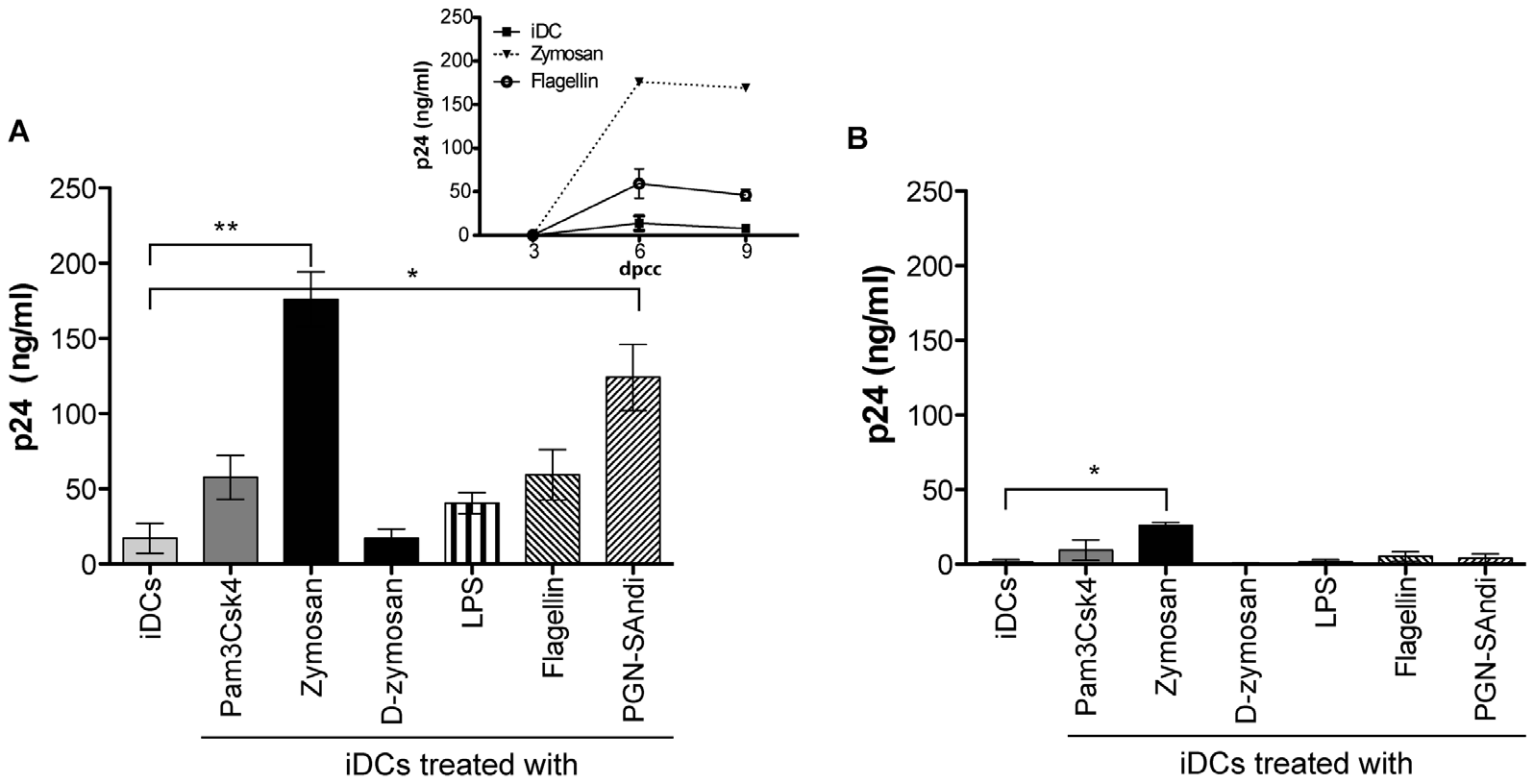
Treatment of DCs with PAMPs promotes *cis*-infection of CD4^+^ T cells with X4 virus. iDCs were left untreated or treated with the indicated PAMPs for 24 hours. Cells were next either left untreated (A) or treated with EFV (B) to block productive infection of DCs prior to loading with NL4-3 for 4 hours at 37°C. Cells were extensively washed and incubated for 16 hours before addition of autologous resting CD4^+^ T cells. Cell-free supernatants were collected at 3, 6 and 9 days following initiation of the co-culture. Data shown represent the mean ± SEM of quadruplicate samples from one representative donor out of four at 6 days following initiation of the co-culture. (*: p<0.05; **: p<0.01). The small insert shows kinetics of virus production for this donor (only iDCs either left untreated or treated with zymosan or flagellin are illustrated).

In the above-described experiments, progeny virus that was measured in the supernatant of co-cultured cells may have been produced by PAMP-treated DCs and then transferred to autologous CD4^+^ T cells (a process known as *cis*-infection) and/or by CD4^+^ T cells after transfer of the input virus by DCs (a process known as *trans*-infection). Thus, in order to define whether the studied PRR ligands affect *cis*- and/or *trans*-infection pathways, similar co-culture experiments were performed but this time by treating DCs with efavirenz (EFV) before virus exposure. This antiviral drug will prevent HIV-1 infection of DCs (i.e. *cis*-infection) without altering their ability to transmit virions located on their surface and/or within their endosomal apparatus (i.e. *trans*-infection) to CD4^+^ T cells. The presence of EFV abrogated almost completely the PAMP-mediated increase in replication of X4-using virus in such co-cultured cells (Figure 4), thus indicating that the enhanced virus production is linked with a productive infection of DCs with HIV-1. Interestingly, EFV treatment had no significant impact on replication of R5 virus in similar co-cultured cells (supporting Figure S1), therefore confirming previous reported data showing that mDCs are refractory to *cis*-infection with R5-using virus.

### PAMP-induced Maturation Process Enhances CXCR4 Expression in DCs and Modifies Intracellular Machinery in CD4^+^ T Cells Resulting in a Higher Susceptibility to Productive Infection with X4 Virus

**Figure 5:**
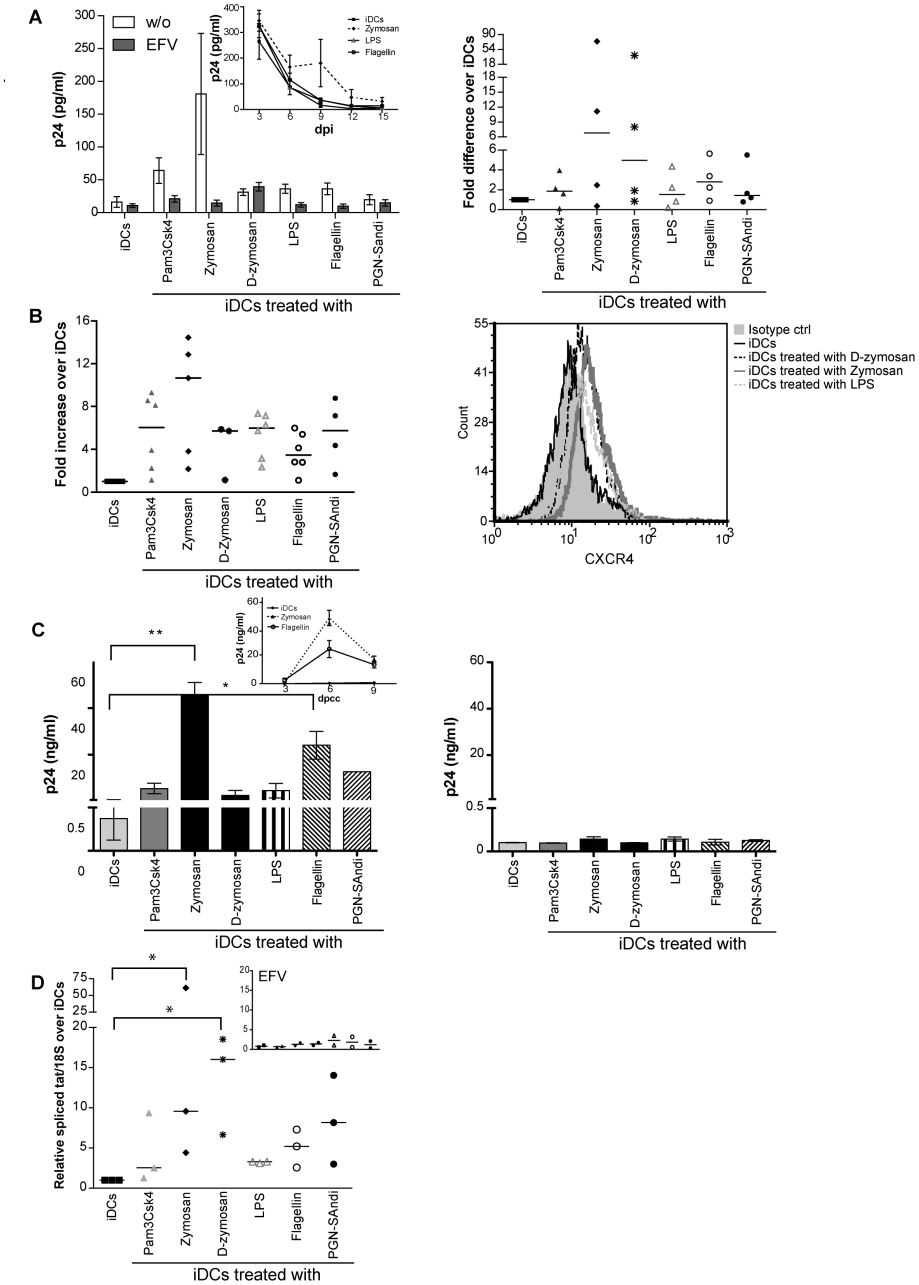
PAMPs enhance CXCR4 expression on DCs and susceptibility to productive infection with X4 virus. (A) iDCs were left untreated or treated for 24 hours with the listed PAMPs. Next, DCs were washed and pretreated, or not, with EFV for 15 minutes, and then exposed to NL4-3 for another 24 hours. Cell-free supernatants were harvested at 3, 6, 9, 12 and 15 days post-infection (dpi). Data depicted in the upper left panel represent the means ± SEM of quadruplicate samples from one representative donor out of four at 6 days post-infection. The small insert shows kinetics of virus production for this donor (only iDCs either left untreated or treated with zymosan, LPS, or flagellin are illustrated). Each point depicted in the right panel represents the mean of quadruplicate samples for each of the four donors tested. The horizontal line represents median results of all four distinct donors. (B) iDCs were left untreated or treated for 72 hours with the indicated PAMPs. Cell surface expression of CXCR4 was analyzed by flow cytometry. Expression of CXCR4 is depicted as the percentage of positive cells multiplied by the mean fluorescence intensity for the treated condition, over that of untreated cells. Each point represents a different donor while the horizontal bar represents the mean of all donors. One representative CXCR4 staining is depicted in the left panel. (C) iDCs were left untreated or treated with the indicated PAMPs for 72 hours to allow CXCR4 surface expression. Cells were next either left untreated (left panel) or treated with EFV (right panel) prior to loading with NL4-3 for 4 hours at 37°C. Cells were extensively washed and incubated for 16 hours before addition of resting CD4^+^ T cells. Supernatants were collected at 3, 6 and 9 days following initiation of the co-culture. Data represents the mean ± SEM of quadruplicates from one representative donor out of four at 6 days following initiation of the co-culture. The small insert on the left panel represents the kinetics of virus infection for this donor. (D) iDCs were either left untreated or treated with the indicated PAMPs for 72 hours. Cells were either left untreated or treated with EFV (small insert) and next incubated with NL4-3 for 72 hours. Total RNA was extracted and the relative amount of spliced *tat* mRNA was evaluated by semi-quantitative RT-PCR. After normalization on 18S ribosomal RNA subunit, the amount of spliced *tat* mRNA in PAMP-treated DCs was expressed as folds of that of untreated cells. Each point represents a different donor and the horizontal bar represents the median of all donors. Asterisks denote statistically significant data (*: p<0.05; **: p<0.01).

The next logical step was to determine whether PAMP-treated human DCs (i.e. mDCs) can effectively be directly infected by X4 virus, a process that can lead to a subsequent *cis*-infection of CD4^+^ T cells. We first monitored virus production in PAMP-treated DCs that were cultured alone in absence of CD4^+^ T cells. As a control, iDCs either left untreated or treated with PAMPs were also subjected to a treatment with EFV to prevent HIV-1 infection. As expected, replication of X4 virus in DCs not treated with PAMPs (i.e. iDCs) is very inefficient (Figure 5A, left panel). Importantly, virus production was slightly augmented following treatment with some PAMPs (e.g. zymosan), although it is mostly likely cytopathic since the kinetics shows a time-dependent decrease of p24 (see small insert). However, when infection of DCs by X4 virus was prevented by EFV, virus replication was diminished in Pam3Csk4-, zymosan-, LPS- and flagellin-treated DCs, therefore suggesting that the observed progeny virus is resulting from a true infection process. Data shown in the right panel of Figure 5A illustrates the high variability between donors. In order to investigate the exact mechanism underlying the increased susceptibility of PAMP-treated DCs to productive infection with X4 virus, we measured the surface expression of HIV-1 coreceptor CXCR4 after exposure to each agonist. Results shown in Figure 5B indicate that surface expression of CXCR4 in DCs is induced by all tested PAMPs, with zymosan being the most potent of them (percentage of positive cells as well as mean fluorescence intensity/MFI for CXCR4 are depicted in Figure S2).

To evaluate the contribution of a higher CXCR4 expression in the increase of *cis*-infection induced by PAMPs, we performed virus transfer experiments with DCs exposed to PAMPs for 3 days to allow an enhanced CXCR4 surface expression. Next, human DCs were inoculated with X4 virus before initiation of the co-culture with autologous resting CD4^+^ T cells. In some conditions, PAMP-treated DCs were treated with EFV before exposure to HIV-1. Results illustrated in Figure 5C confirm that the PAMP-mediated enhancement in replication of X4 virus is due to a *cis*-infection process, which relies itself on productive HIV-1 infection of DCs. We next assessed productive infection of PAMP-treated DCs by quantifying tat mRNA levels. We found higher amounts of tat mRNA in DCs that were treated with all PAMPs tested (Figure 5D). No tat mRNA could be detected in presence of EFV, which corroborates that PAMP-treated DCs are productively infected with X4 virus.

**Figure 6:**
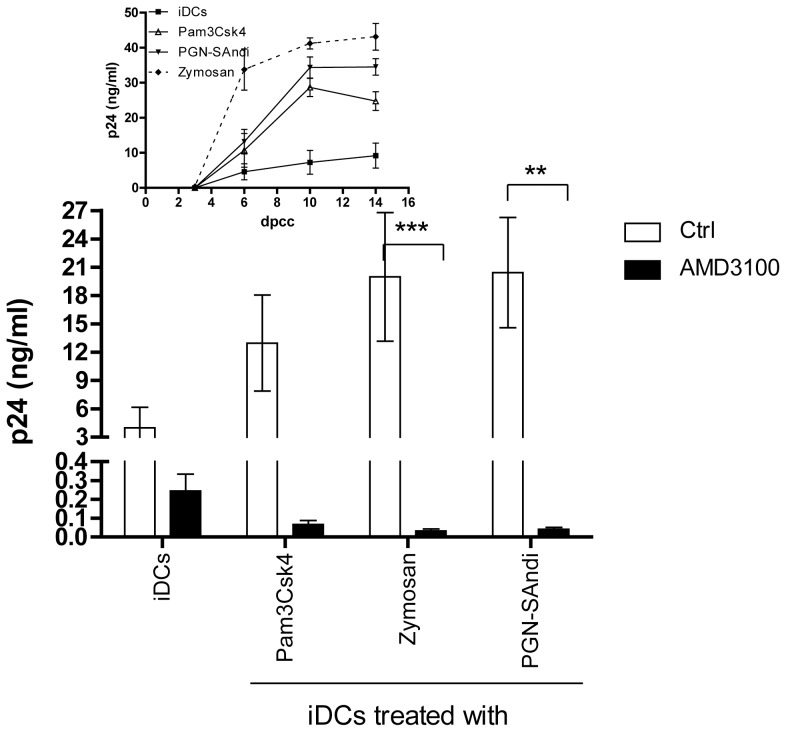
Replication of X4 virus in DC-T cell co-cultures in presence of different PAMPs and AMD3100. iDCs were first either left untreated or treated for 24 hours with the listed PAMPs. Next, cell were extensively washed 3 times with PBS and treated for 30 min with AMD3100 (20 µg/ml), washed again extensively to remove the residual drug and inoculated with NL4-3 (10 ng of p24 per 10^5^ cells) for 1 hour at 37°C. Cells were again extensively washed before initiation of co-culture with autologous resting CD4^+^ T cells. Cell-free supernatants were harvested at different days following initiation of the co-culture (day 10 is depicted). The viral content was assessed by performing a p24 ELISA test. Data shown represent the means ± SEM of quadruplicate samples from two distinct donors. Asterisks denote statistically significant data (**: p<0.01). The small insert shows kinetics of virus production for one donor.

To demonstrate the importance of the PAMP-mediated enhancement in CXCR4 surface expression to the noticed increase in HIV-1 replication in DCs, we performed experiments with the bicyclam AMD3100. This compound blocks HIV-1 entry and membrane fusion via the CXCR4 coreceptor. Data from Figure 6 indicate that the PAMP-directed increase in HIV-1 production is no longer detected in DC-T cell co-cultures. It can be seen that virus production is almost completely abrogated in presence of AMD3100. These results when combined with our previous observations indicate that the observed PAMP-directed effect is at least partly due to DC maturation, which allows productive HIV-1 infection of DCs through a CXCR4- and fusion-dependent process.

Altogether, our results suggest that maturation of human DCs with dectin-1/TLR2 and NOD2 ligands allows productive infection of such cells with X4 virus. A subsequent physical contact between HIV-1-infected mDCs and autologous CD4^+^ T cells is sufficient *per se* to promote virus replication in the latter cell population.

## Discussion

In the present work, we demonstrate that once iDCs are in contact with some specific PAMPs they become susceptible to infection with X4 isolates of HIV-1 and their maturation profile sensitizes CD4^+^ T cells that are resting at the beginning of the co-culture, rendering the latter cell population more permissive to productive virus infection. All experiments described here were performed with monocyte-derived DCs. It is known that circulating monocytes represent an important source of conventional DCs under both inflammatory and steady-state conditions [31]. By using monocyte-derived DCs, which is currently the most common and convenient method to study HIV-1 transfer and infection, one can obtain an homogeneous DC population in an immature state that shares common characteristics with mucosal tissue-homing DCs. In contrast, circulating DCs isolated directly from peripheral blood represent heterogeneous populations, which include DC precursors from bone marrow, differentiated plasmacytoid, conventional iDCs and mDCs that have captured antigen in peripheral tissues and are re-entering circulation to mount an adaptive immune response (reviewed in [32], [33]). Based on this information, we thought it was more appropriate to use monocyte-derived DCs in order to obtain a more homogenous population.

When resident-tissue iDCs are exposed to a given pathogen or microbial-derived products, they enter a maturation process and rapidly migrate in SLOs to initiate an adaptive immune response [34]. The migratory capacity of DCs is tightly linked with their maturation state and involves expression of chemokine receptors CCR7 and CXCR4. We report here that all PAMPs tested, with the exception of D-zymosan, induced a significant increase in CCR7 expression in DCs, while all of the stimuli enhanced, by at least 4 folds, the surface expression of CXCR4 when compared to iDCs. This feature might benefit X4 virions present in SLOs and influence viral transmission in neighbouring CD4^+^ T cells. The initiation of the immune response requires a physical contact between DCs and antigen-specific T cells within the complex environment of the lymph node. Knowing the low frequency of this event (i.e. one in 10^5^ to 10^6^), DCs must rapidly scan a high number of CD4^+^ T cells to establish these rare cognate interactions [35]. The scanning is promoted by successive extension and retraction of their dendrites that can reach a speed of 60 µm/min. This phenomenon may also favor the capture of free circulating HIV-1 particles present in SLOs. Interestingly, we found that iDCs treated with zymosan, PGN-SAndi and D-zymosan acquire a morphology characterized by long dendrites, which is maintained for at least 24 hours post-exposure. This feature can most likely promote HIV-1 capture, and may partly explain the noticed *cis-*infection of CD4^+^ T cells with X4 virus. During the first hours following their entry in SLOs, CD4^+^ T cells establish brief contacts with DCs (<5 min) that might be sufficient to allow cell-to-cell transmission of HIV-1 [36], [37], [38]. It can thus be proposed that virus-infected mDCs (in which the maturation process is induced by some microbial-derived products) can transmit HIV-1 to numerous surrounding CD4^+^ T cells, a phenomenon that can ultimately enhance the pool of virus reservoirs.

We provide evidence that a contact between mDCs and CD4^+^ T cells is required to achieve the zymosan- and PGN-Sandi-mediated enhancing effect on HIV-1 spreading from DCs toward autologous CD4^+^ T cells. Surprisingly, PAMPs that can induce a full maturation profile in DCs exert a lower impact on CD4^+^ T cell sensitization required for HIV-1 replication. Accordingly, it appears that the spreading of X4 virus is not only influenced by the ability of mDCs to sensitize CD4^+^ T cells but also by the capability of mDCs to allow a complete viral life cycle (i.e. productive virus infection). The importance of these two events is highlighted with the use of the dectin-1 ligand, D-zymosan. Indeed, D-zymosan-treated DCs contain a high level of spliced *tat* mRNA, but this is not correlated with HIV-1 production in the DC-T cell co-culture. This might partially result from a reduced ability of D-zymosan-treated DCs to properly sensitize CD4^+^ T cells to productive HIV-1 infection. Our data fully support this hypothesis since DCs treated with D-zymosan express less maturation markers than zymosan-treated cells but express similar level of IL-1β, IL-23p19 and IL-12p40 mRNAs, therefore suggesting that direct priming through T-cell and co-stimulatory receptors might be weaker. The degree of T-cell sensitization is important to further amplify HIV-1 replication in the co-culture. Our results show that the phenotype of PAMP-induced DC maturation appears to be different even if signaling pathways downstream of PRRs are mostly common. The maturation profile may be different in order to respond properly and adapt the response to a specific type of pathogen.

Signal transduction pathways that can drive DC maturation might influence HIV-1 post-entry steps and impact viral transfer. *Cis*- and *trans*-infection processes of R5- and X4-tropic HIV-1 from DCs to CD4^+^ T cells are two dissociable events differently affected by the maturation state of DCs. Some previous studies have already demonstrated that engagement of TLR2, TLR3, TLR4, TLR7/8 and TLR9 by PAMPs or their synthetic analogs like Pam3Csk4, polyI:C, R848 or CPGs differently influence HIV-1 transfer of R5-using viruses from mDCs to activated CD4^+^ T cells or T cell lines [27], [39], [40], [41], [42], [43]. It has been already established that a brief treatment of iDCs with TLR2 ligans (i.e. 1 hour) enhanced HIV-1 transfer and infection of activated CD4^+^ T cells by CCR5 virus [26]. Similarly, Grighuis and co-workers have recently demonstrated that HIV-1 exploits TLR8- and DC-SIGN-mediated signalling cascades to achieve productive infection of DCs [44]. We found that most of the DC-mediated transmission of R5-using virions toward CD4^+^ T cells is also due to a *trans*-infection mode. Indeed, it has been reported that the *cis*-infection process of R5-using variants is abolished in mDCs mainly through an inhibition of HIV-1 fusion (due to a CCR5 depletion), reverse transcription and transcription [39]. The reduced viral production in our co-culture system may also result from an inability of mDCs to modulate CCR5 expression in CD4^+^ T cells. In fact, we could not detect any increase in CCR5 surface levels in CD4^+^ T cells, thus suggesting that the sensitizing effect mediated by PAMP-treated DCs is not sufficient to increase CCR5 expression. Viral replication within DCs is not proficient as in effector CD4^+^ T cells partly because of multiple cellular restriction factors (e.g. SAMHD1), but it appears to be sufficient to allow HIV-1 transmission to CD4^+^ T cells. In fact, the use of ultrapure Pam3Csk4, LPS, flagellin, D-zymosan and PGN-SAndi revealed that increased viral production was mainly due to an enhanced *cis*-infection process. Knowing that engagement of the CLR DC-SIGN induces signal transduction events promoting HIV-1 replication in DCs [45], [46], we postulate that engagement of the CLR dectin-1 may also render these cells susceptible to viral replication. The CLR downstream signal involved the activation of the tyrosine kinase Syk which is coupled to inflammasome activation and IL-1β secretion [47]. We previously reported that Syk is important for an efficient HIV-1 infection of iDCs and subsequent virus transfer to CD4^+^ T cells [48]. Given that dectin-1 and DC-SIGN interact together to mediate signal transduction events in DCs [49], that zymosan can engage both dectin-1 and DC-SIGN, and that HIV-1 infection of DCs is promoted by an interaction between virus-associated gp120 and DC-SIGN [44], it can be proposed that the zymosan-directed enhancement in virus replication in DCs is due to both dectin-1 and DC-SIGN. Similarly, the activation of NOD2 also leads to inflammasome activation. Therefore, it seems that when DCs are primed by agonists triggering the activation of inflammasomes, such cells lose restriction factors impairing HIV-1 life cycle. It has been recently reported that SAMHD1 represents an important restriction factor for HIV-1 in myeloid cells and resting CD4^+^ T cells, which acts by controlling the intracellular pool of dNTPs [50], [51]. Virus replication can also be limited by other cellular factors involved in the maintenance of steady-state conditions and a basal metabolism, which are present in iDCs and resting CD4^+^ T cells [52], [53]. Interestingly, a recent work has shown that a physical contact between DCs and CD4^+^ T cells is adequate to reduce SAMHD1 activity and allow HIV-1 infection of resting CD4^+^ T cells [54]. Interestingly, when iDCs were first exposed to viral particles and 24 hours later put in presence of PAMPs like zymosan or PGN-SAndi, the same up-regulatory effect on DC-mediated *cis*-infection of CD4^+^ T cells by X4 virions was seen (Figure S3), thus confirming that treatment of iDCs with some PAMPs is enough to reduce the limiting effect exerted by restriction factors.

Overall our observations allow us to propose the following hypothetical model describing the physiological effect of PAMPs on X4-tropic expansion through DCs. Microbial translocation and opportunistic infections, two distinctive features of HIV-1 infection, will result in an increase of circulating microbial products and a penetration into deeper tissues. These microbial products will be recognized by iDCs found in the periphery through PRRs and this process will lead to their maturation. We have shown that most PAMPs can induce maturation of DCs and enhance CCR7 expression which will allow DCs to migrate to SLOs. The expression of CXCR4 is also increased following an exposure to most PAMPs and this will result in a higher susceptibility of mDCs to productive infection with X4-using HIV-1 strains. Moreover, PAMP-treated DCs (particularly those exposed to bacterial- and yeast-derived products) have the capacity to sensitize CD4^+^ T cells for a productive infection with HIV-1.

## Experimental Procedures

### Ethics Statement and Cell Culture

Human peripheral blood mononuclear cells were obtained from anonymous and paid, healthy volunteer donors that were specifically solicited for provision of these samples. Healthy subjects signed an informed consent form approved by the Institutional Review Board (IRB) of the Centre Hospitalier Universitaire (CHU) de Québec. The current research project was also approved by our IRB. No research was performed outside of Canada. To obtain monocyte-derived iDCs, total peripheral blood mononuclear cells were first isolated from peripheral blood from healthy donors by Ficoll centrifugation according to the manufacturer’s protocol (Wisent, St-Bruno, Canada). Monocytes were then purified using an immunomagnetic CD14-positive selection kit (Stem Cells Technologies Inc., Vancouver, Canada). Purified CD14^+^ cells were cultured in complete RPMI medium supplemented with GM-CSF (1000 U/mL) and IL-4 (200 U/mL) for 6 days to generate iDCs and their purity was assessed by flow cytometry as previously described [55]. Autologous resting CD4^+^ T cells were isolated using a negative selection kit according to the manufacturer’s instructions (Stem Cells Technologies Inc., Vancouver, Canada), and cultured in complete RPMI medium until use. Human embryonic kidney 293T cells that were used for transient transfection experiments were cultured in complete DMEM medium. HEK-blue™ IFNα/β cells, which were used for measuring the level of type-I IFN, were purchased from InvivoGen (San Diego, USA) and maintained in complete DMEM media supplemented with 100 µg/ml zeocin and 10 µg/ml blasticidin (both from Invivogen, San Diego, USA).

### Antibodies and Reagents

PRR agonists were all purchased from Invivogen (San Diego, USA) and include Pam3Csk4 (a synthetic tripalmitoylated lipopeptide that mimics the acylated amino-terminus of bacterial lipoproteins; a TLR2 agonist), ultra-purified lipopolysaccharide (LPS) from *Escherichia coli* (TLR4 agonist), purified flagellin from *Salmonella typhimurium* (TLR5 agonist), ultrapure PGN-Sandi (an insoluble peptidoglycan from *Staphylococcus aureus*; a NOD2 agonist), zymosan (cell wall from *Saccharomyces cerevisiae*; a TLR2 and dectin-1 agonist), depleted zymosan (D-zymosan) (hot-alkali-treated zymosan; a dectin-1 agonist), and poly(I:C) (a synthetic analog of dsRNA; a TLR3 agonist). IL-4 was purchased from Biolegend (San Diego, USA) and granulocyte macrophage-colony stimulating factor (GM-CSF) from Genscript (Piscataway, USA). Efavirenz (EFV) was obtained from the NIH AIDS Research and Reference Reagent Program (Germantown, USA). The culture media used were RPMI-1640 (Wisent, St-Bruno, Canada) and DMEM (Invitrogen, Burlington, Canada) supplemented with 10% heat-inactivated fetal bovine serum (FBS) (Wisent, St-Bruno, Canada), 100 U/mL penicillin-streptomycin (Invitrogen, Burlington, Canada) and 100 U/mL primocin (Invivogen, San Diego, USA). Hybridomas producing 183-H12-C5 and 31-90-25, two antibodies specific for the HIV-1 viral core protein p24 and used for ELISA, were supplied by the NIH AIDS Research and Reference Reagent Program and ATCC (Manassas, USA), respectively. Antibodies were purified from culture supernatants using mAbTrap protein G affinity columns according to the manufacturer’s recommendations (GE Healthcare, Mississauga, Canada). The following R-Phycoerythin (R-PE)- or fluorescein (FITC)-conjugated antibodies and their corresponding isotype-matched controls that were used in flow cytometry studies were purchased from BD Biosciences (Mississauga, Canada): antibodies specific for CD3 (clone UCHT1), HLA-DR (clone L243), CD80 (clone L307.4), CD86 (clone 2331), CD25 (clone 2A3), CD54 (clone HA58), and CXCR4 (clone 12G5). Antibodies directed against CCR7 (clone150503) and CCR5 (clone 3A9) were obtained from R&D Systems (Minneapolis, USA) while anti-CD14 (clone 61D3), -CD83 (clone HB15e) and -CD209 (DC-SIGN, clone eB-h209) were purchased from eBioscience (San Diego, USA).

### Production of Viral Stocks

Virus particles were produced by calcium-phosphate transfection of 293T cells [56] using 40 µg of pNL4-3 (X4) [57] or pNL4-3/*Balenv* (R5) [58]. In the latter infectious molecular clone of HIV-1, the *env* gene of the X4 (T)-tropic NL4-3 strain has been replaced with that of the R5 (macrophage)-tropic Bal strain. Virus preparations were filtered through a 0.22 µm cellulose acetate filter, and quantified by an in-house double-antibody sandwich ELISA specific for the p24 protein [59].

### Flow Cytometric Analysis of Activation Markers in DCs and CD4^+^ T Cells

iDCs were either left untreated or treated for 72 hours with one of the PRR agonist. In such experiments, Fc receptors were blocked using normal human serum for 15 minutes at 4°C. Next, cells (1×10^5^) were stained with 50 ng of selected antibodies (specific for DC-SIGN, CD83, CCR7, CD80, or CD86) for 30 minutes and washed twice in ice-cold phosphate-buffered saline (PBS) containing 5% FBS. Resting CD4^+^ T cells were cultured alone or in the presence of autologous DCs (previously treated with a PRR agonist for 24 hours) at a 3∶1 ratio. After 6 days of co-culture, cells were stained with antibodies against CD25, CD54 and HLA-DR, as described for the analysis of DC activation markers. In these experiments, CD3-expressing cells were gated in order to analyze only T cells. Non-specific staining was monitored using isotype-matched control irrelevant antibodies. Cells were fixed in 2% paraformaldehyde for 30 minutes at 4°C, and data were collected using an EPICS ELITE ESP apparatus (Coulter Electronics, Burlington, Canada). Data were analyzed with the FCS express software (De Novo Software, Los Angeles, USA).

### Semi-quantitative Real-time PCR Analysis of Cytokine Expression

iDCs (1×10^6^) were either left untreated or treated for 4 or 24 hours with one of the PRR agonist. Total RNA was isolated using the IllustraRNAspin Mini RNA Isolation Kit (GE Healthcare, Mississauga, Canada), and RNA was reverse-transcribed using Superscript III RT (Invitrogen, Burlington, Canada). The relative amount of cDNA for IL-1β, IL-6, IL-12p40 and IL-23 was evaluated using Power SYBR®Green PCR Master Mix (Applied Biosystem, Carlsbad, USA) in a 7500 real-time PCR apparatus (Applied Biosystem, Carlsbad, USA). 18S ribosomal RNA subunit was used as an internal endogenous loading calibrator in these 2^-ΔΔCT^ experiments. Sequences of the primers are illustrated in used can be found as a Supporting Information File S1.

### Quantification of Type-I IFN

iDCs were either left untreated or treated for 6 hours with one of the PRR agonist. Next, levels of type-I IFN were determined in cell-free supernatants using HEK-Blue™ IFNα/β cells according to the manufacturer's protocol (InvivoGen). HEK-Blue™ IFNα/β cells are stably transfected with a SEAP promoter gene under the control of the IFNα/β-inducible ISG54 promoter, and thus allow the detection of bioactive IFNα and IFNβ by monitoring the activation of the ISGF3 pathway. A standard curve of IFNα ranging from 1 to 250 units/ml was used to quantify the amounts of type-I IFN released in the culture medium.

### T-cell Proliferation Assay

Resting CD4^+^ T cells were labeled with the carboxyfluorescein diacetate succinimidyl ester (CFSE) (2 µM) as described by the manufacturer (Invitrogen) and kept in culture media for 24 hours at 37°C. Next, iDCs were either left untreated or treated with the indicated PAMPs for 24 hours, washed and co-cultured with CFSE-labelled autologous resting CD4^+^ T cells (ratio 1∶10) for 6 days at 37°C. Finally, cells were washed in PBS, fixed in 2% paraformaldehyde and analyzed by flow cytometry. Data were analyzed with the FCS express software.

### HIV-1 Infection of DC-T Cell Co-cultures

iDCs (3×10^4^ cells in 100 µL culture medium) were either left untreated or treated with one of the studied PRR agonists (1 µg/mL for Pam3CSK4, flagellin, PGN-SAndi and poly(I:C); 5 µg/mL for zymosan and D-zymosan; and 10 ng/mL for ultrapure LPS) for 24 hours in order to induce the maturation process. Cell viability was assessed with a MTS assay as described by the manufacturer (Promega, Madison, USA). Following DC maturation, cells were washed twice with PBS and exposed to a virus preparation (10 ng of p24 per 1×10^5^ cells) for the indicated period (1 or 4 hours) at 37°C. Where indicated, efavirenz (EFV; 25 nM) was added 15 minutes prior to virus exposure and maintained during virus pulse in order to block productive infection of DCs. Next, cells were washed twice with PBS to remove free viruses and EFV when present. Autologous resting CD4^+^ T cells were immediately co-cultured with DCs (3∶1 ratio) in a final volume of 200 µL in complete RPMI-1640 medium in 96-well plates. In some experiments, DCs were incubated for an additional 16 or 72 hours after washes, before adding CD4^+^ T cells. Cell-free co-culture supernatants were harvested at 3, 6 and 9 days following initiation of the co-culture and kept at –20°C until analysis. Then, p24 assays (see above section) were performed to quantify the virus content.

### HIV-1 Infection of DCs

iDCs (5×10^4^ cells in 100 µL culture medium) were either left untreated or treated with one of the studied PRR agonists for 24 hours to induce DC maturation. Next, mDCs were washed twice with PBS and exposed to virus preparations (10 ng of p24 per 1×10^5^ cells) for 24 hours at 37^o^C. In some studies, EFV (25 nM) was added 15 minutes prior to virus addition and maintained during virus pulse in order to block productive infection of DCs. Then, cells were washed twice with PBS to remove free viruses, resuspended in culture medium and incubated at 37°C. Cell-free supernatants were harvested at 3, 6, 9, 12 and 15 days post-infection and kept at −20°C until analysis. Then, p24 assays were performed to quantify the viral content.

### HIV-1 Infection in Resting CD4^+^ T Cells Cultured with mDCs

Resting CD4^+^ T cells were inoculated with NL4-3 (10 ng of p24 per 1×10^5^ cells at a concentration of 10×10^6^ cells/ml) for 1 hour at 37°C and washed three times with PBS. Cells were then resuspended in culture medium at a final concentration of 2×106 cells/ml and incubated at 37°C for 48 hours. Cells were next seeded in 96-well plates containing mDCs that were previously treated for 24 hours with one of the listed PRR agonists. Cell-free supernatants were harvested at 3, 6 and 9 days following initiation of the co-culture and the p24 content was quantified by ELISA.

### Detection of HIV-1 mRNA

iDCs (5×10^5^ cells) were either left untreated or treated for 72 hours with one of the studied PRR agonists. Cells were washed with PBS, treated or not with EFV (25 nM) to block productive infection and exposed to NL4-3 (10 ng of p24 per 1×10^5^ cells). After 72 hours, total RNA was extracted as mentioned above for cytokine gene expression analysis. The relative amount of spliced *tat* mRNA was evaluated by real-time PCR analysis using a relative standard curve method. The sequences of the primers used can be found in Supporting Information File S1.

### Statistical Analysis

Means of raw data for Figures 1A, 4A, 4B, 5C, 5D and 6 were compared using Kruskal-Wallis non-parametric analysis followed by Dunn’s post-test, whereas means of raw data for Figures 1B and 3 were compared using repeated measure ANOVA with Dunnett’s post-test. For the data shown in Figure 2, statistical analyses were made using the percentage of positive cells or the mean fluorescence intensity by using a Wilcoxon test. *P* values lower than 0.05 were deemed significant. Calculations were performed using GraphPad Prism version 5.0 software.

## Supporting Information

Figure S1 **Propagation of R5 virus in DC-T cell co-cultures is modulated at a lower extent by PAMPs.** iDCs were left untreated or treated with the indicated PAMPs for 24 hours. Cells were next either left untreated (left panels) or treated with 25 nM EFV (right panels) to block productive infection of DCs prior to loading with NL4-3/*Balenv* for 4 hours at 37°C. Cells were extensively washed and incubated for 16 hours before addition of autologous resting CD4^+^ T cells. Cell-free supernatants were collected at 3, 6 and 9 days following initiation of the co-culture and kept at –20^o^C until assayed for the p24 content. Data depicted in the upper panels represent the means ± SEM of quadruplicate samples from one donor at 6 days following initiation of the co-culture. The small insert shows kinetics of virus production for this donor (only iDCs either left untreated or treated with zymosan or flagellin are illustrated). Each point depicted in the lower panels represents the mean of quadruplicate samples for each of the different donors tested and the horizontal line represents median results of all the different donors tested. Asterisks denote statistically significant data (*: p<0.05; **: p<0.01).
(TIF)

Figure S2 **Expression of DC maturation markers following treatment with PAMPs.** A) iDCs were either left untreated or treated for 72 hours with the indicated PAMPs. Thereafter, cell surface expression of DC-SIGN, CD83, CD86, CD80 and CCR7 was evaluated by flow cytometry. Table 1 represent the means±SEM of the percentage of positive cells while table 2 depicts the means±SEM of the mean fluorescence intensity for all donors tested (ranging from 6 to 15, as indicated by the N value). B) CXCR4 staining (open histogram with black line) compared to isotype control (fill histogram) for all conditions tested is shown for one representative donor.
(TIF)

Figure S3 **iDCs first exposed to X4 virus and next to PAMPs display a similar capability to promote** ***cis*** **-infection of resting CD4^+^ T cells.** iDCs were first incubated for 24 hours at 37^o^C with NL4-3 (10 ng of p24 per 1×10^5^ cells). Next, iDCs were either left untreated or treated with the indicated PAMPs for 24 hours. Cells were washed twice with PBS, resuspended in culture medium and incubated at 37°C. Autologous resting CD4^+^ T cells were added 4 days later at a 3∶1 ratio in a final volume of 200 µL in complete RPMI-1640 medium in 96-well plates. Cell-free supernatants were collected at 3, 6 and 9 days following initiation of the coculture and kept at -20^o^C until assayed for the p24 content. Data depicted in the upper panel represent the means ± SEM of quadruplicate samples from one donor out of eight at 6 days following initiation of the coculture. The small insert shows kinetics of virus production for this donor (only iDCs either left untreated or treated with zymosan or flagellin are illustrated). Each point depicted in the lower panel represents the mean of quadruplicate samples for each of the different donors tested and the horizontal line represents median results of all the different donors tested.
(TIF)

File S1 **Supporting materials and methods.**
(DOCX)
