# Supplementary figures and images for: Dectin-1/TLR2 and NOD2 Agonists Render Dendritic Cells Susceptible to Infection by X4-Using HIV-1 and Promote cis-Infection of CD4+ T Cells
Source: PLoS One. 2013 Jul 2;8(7):e67735. doi: 10.1371/journal.pone.0067735 (PMC3699635; doi:10.1371/journal.pone.0067735)

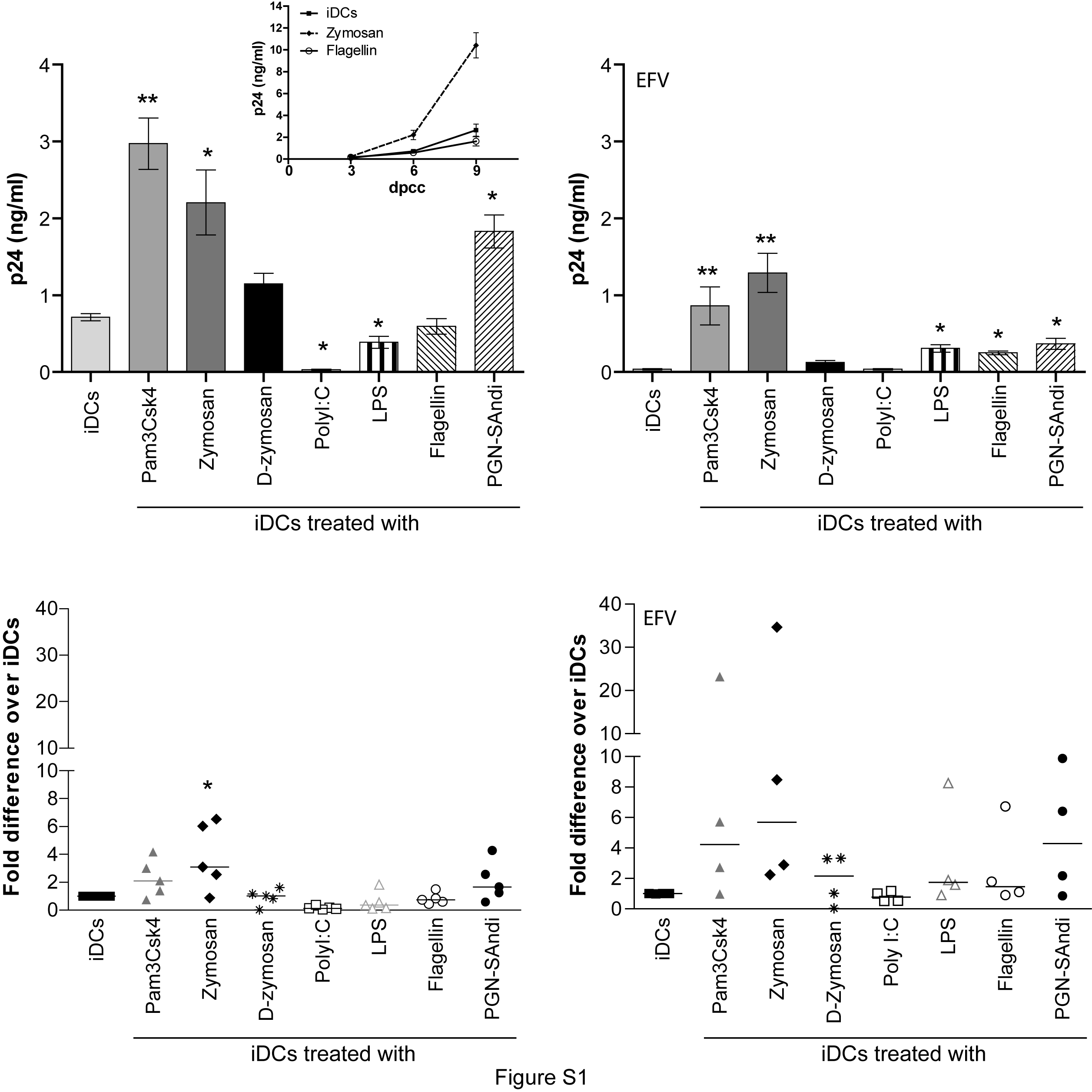

Supplement: Figure S1 — Propagation of R5 virus in DC-T cell co-cultures is modulated at a lower extent by PAMPs. iDCs were left untreated or treated with the indicated PAMPs for 24 hours. Cells were next either left untreated (left panels) or treated with 25 nM EFV (right panels) to block productive infection of DCs prior to loading with NL4-3/Balenv for 4 hours at 37°C. Cells were extensively washed and incubated for 16 hours before addition of autologous resting CD4+ T cells. Cell-free supernatants were collected at 3, 6 and 9 days following initiation of the co-culture and kept at –20oC until assayed for the p24 content. Data depicted in the upper panels represent the means ± SEM of quadruplicate samples from one donor at 6 days following initiation of the co-culture. The small insert shows kinetics of virus production for this donor (only iDCs either left untreated or treated with zymosan or flagellin are illustrated). Each point depicted in the lower panels represents the mean of quadruplicate samples for each of the different donors tested and the horizontal line represents median results of all the different donors tested. Asterisks denote statistically significant data (*: p<0.05; **: p<0.01). (TIF) [file pone.0067735.s001.tif]

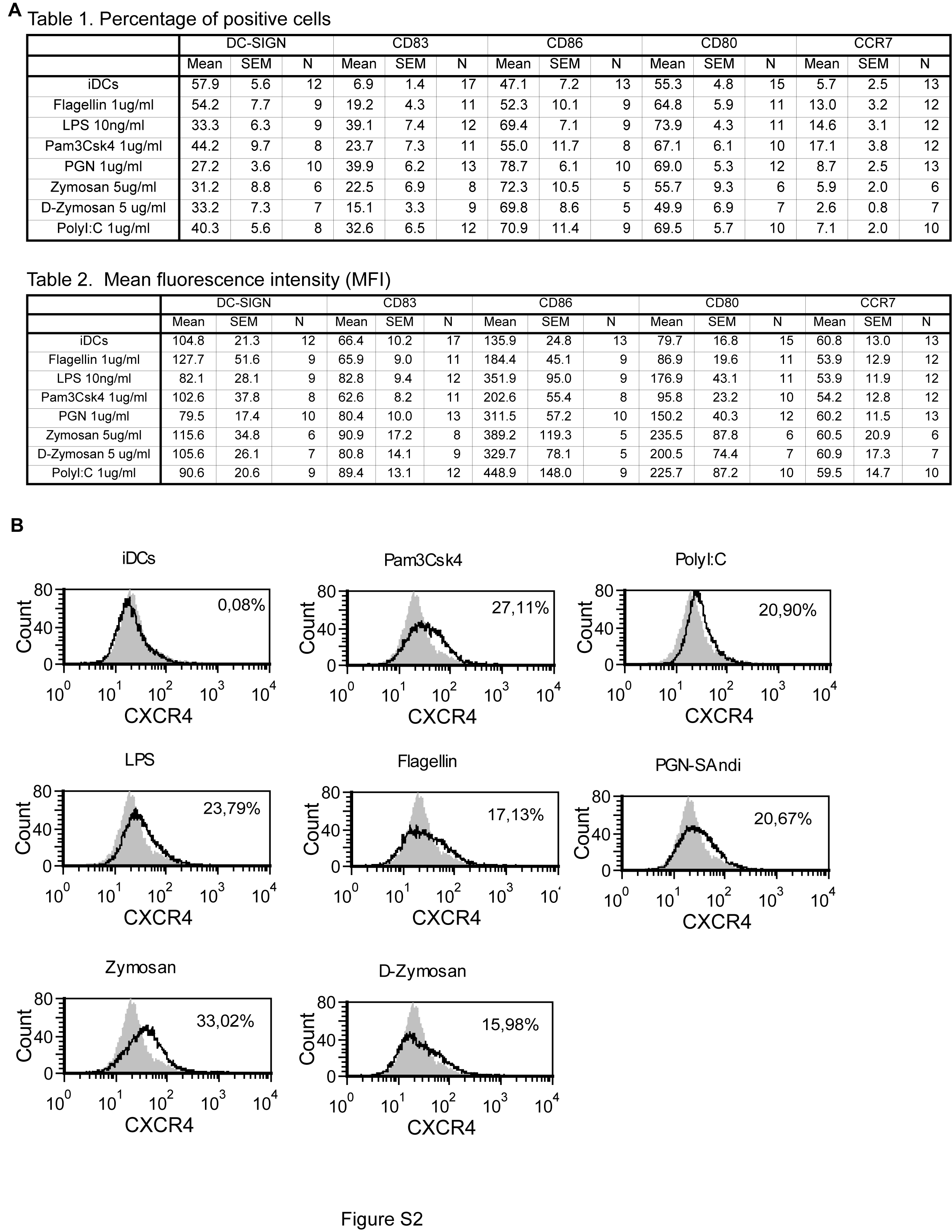

Supplement: Figure S2 — Expression of DC maturation markers following treatment with PAMPs. A) iDCs were either left untreated or treated for 72 hours with the indicated PAMPs. Thereafter, cell surface expression of DC-SIGN, CD83, CD86, CD80 and CCR7 was evaluated by flow cytometry. Table 1 represent the means±SEM of the percentage of positive cells while table 2 depicts the means±SEM of the mean fluorescence intensity for all donors tested (ranging from 6 to 15, as indicated by the N value). B) CXCR4 staining (open histogram with black line) compared to isotype control (fill histogram) for all conditions tested is shown for one representative donor. (TIF) [file pone.0067735.s002.tif]

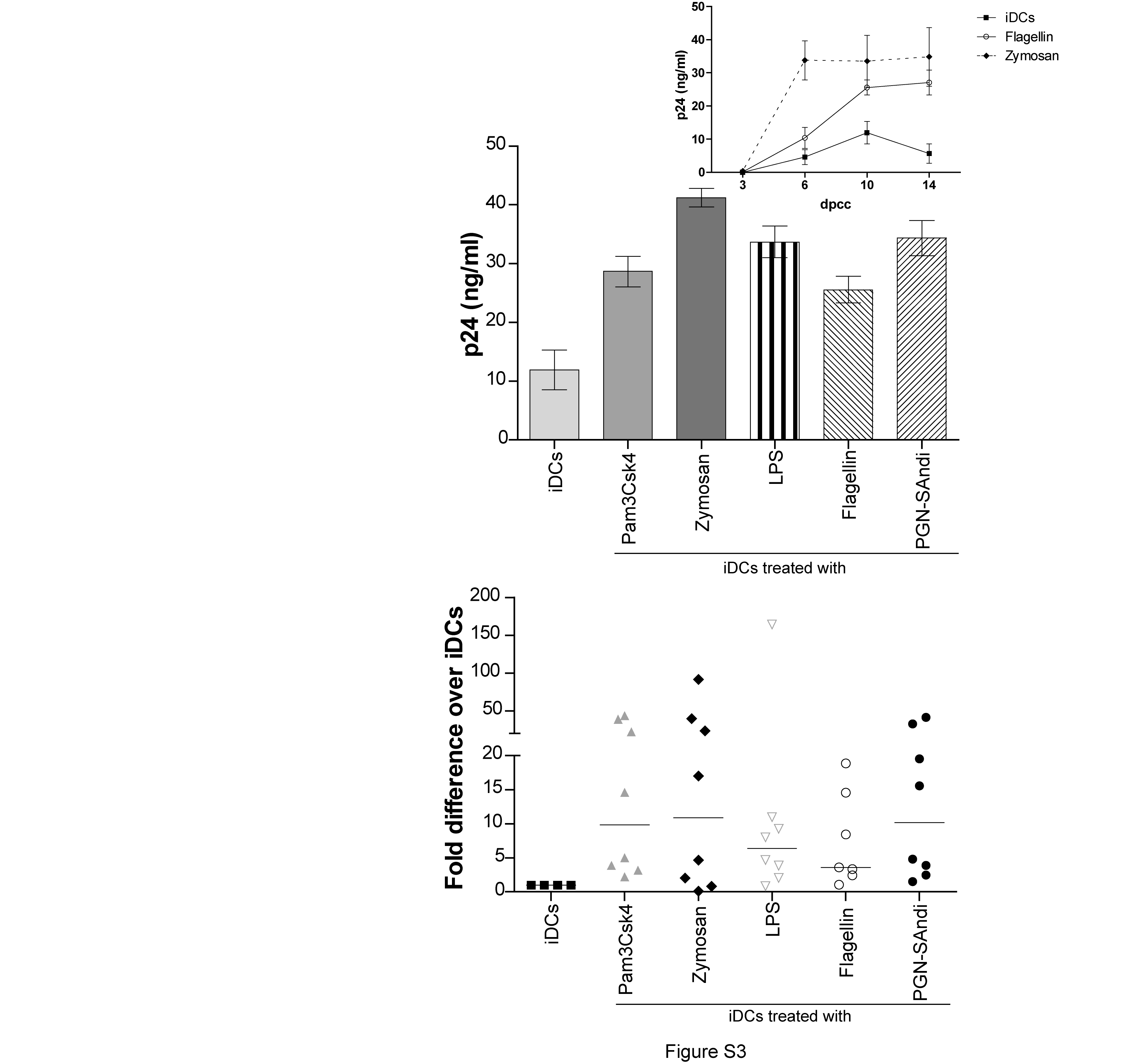

Supplement: Figure S3 — iDCs first exposed to X4 virus and next to PAMPs display a similar capability to promote cis -infection of resting CD4+ T cells. iDCs were first incubated for 24 hours at 37oC with NL4-3 (10 ng of p24 per 1×10^5^ cells). Next, iDCs were either left untreated or treated with the indicated PAMPs for 24 hours. Cells were washed twice with PBS, resuspended in culture medium and incubated at 37°C. Autologous resting CD4+ T cells were added 4 days later at a 3∶1 ratio in a final volume of 200 µL in complete RPMI-1640 medium in 96-well plates. Cell-free supernatants were collected at 3, 6 and 9 days following initiation of the coculture and kept at -20oC until assayed for the p24 content. Data depicted in the upper panel represent the means ± SEM of quadruplicate samples from one donor out of eight at 6 days following initiation of the coculture. The small insert shows kinetics of virus production for this donor (only iDCs either left untreated or treated with zymosan or flagellin are illustrated). Each point depicted in the lower panel represents the mean of quadruplicate samples for each of the different donors tested and the horizontal line represents median results of all the different donors tested. (TIF) [file pone.0067735.s003.tif]
