# Supplementary material for: Dectin-1/TLR2 and NOD2 Agonists Render Dendritic Cells Susceptible to Infection by X4-Using HIV-1 and Promote cis-Infection of CD4+ T Cells
Source: PLoS One. 2013 Jul 2;8(7):e67735. doi: 10.1371/journal.pone.0067735 (PMC3699635; doi:10.1371/journal.pone.0067735)
Supplement: File S1 — Supporting materials and methods. (DOCX) [file pone.0067735.s004.docx]

**Supporting Materials and Methods**

*Primers used*

IL-1β forward:GGACAAGCTGAGGAAGATGC

IL-1β, reverse:TCGTTATCCCATG-TGTCGAA

IL-6, forward: GAAAGCAGCAAAGAGGCACT

IL-6, reverse: TTTCACCAG-GCAAGTCTCCT

IL12p40,forward:AAGGAGGCGAGGTTCTA-AGC

IL12p40, reverse: GCAGGTGAAACGTCCAGAAT

IL-23p19,forward:TTCTCT-GCTCCCTGATAGCC

IL-23p19: GACTGAGGCTTGGAATCTGC

Ribosomal18Ssubunit,forward:tagagggacaagtggcgttc

Ribosomal 18S subunit, reverse cgctgagccagtcag-tgt

tat, forward: GAAGCATCCAGGAAGTCAGC

tat, reverse: CTATTCCTTCGGGCCTG-TC

*Virus replication in DCs first exposed to HIV-1 and then to the studied PAMP agonists (Figure S2).* iDCs (5 x 10^4^ cells in 100 µL culture medium) were exposed to HIV-1 (10 ng of p24 per 1 x 10^5^ cells) for 24 hours at 37^o^C. In some studies, EFV (25 nM) was added 15 minutes prior to virus addition and maintained during virus pulse in order to block productive infection of DCs. Cells were then extensively washed with PBS and treated for 24 hours with the listed PAMPs. Following maturation, cells were washed twice with PBS, resuspended in culture medium and incubated at 37°C. Autologous resting CD4^+^ T cells were added 4 days later at a 3:1 ratio in a final volume of 200 µL in complete RPMI-1640 medium in 96-well plates. Cell-free culture supernatants were harvested at 3, 6, 10 and 14 days following initiation of the co-culture and kept at -20^o^C until assayed for the p24 content.
